# Low Calcium–High Magnesium Krebs–Henseleit Solution Combined with Adenosine and Lidocaine Improved Rat Aortic Function and Structure Following Cold Preservation

**DOI:** 10.3390/medicina60081284

**Published:** 2024-08-09

**Authors:** Aryadi Arsyad, Geni K. R. Lembang, Sesilia L. Linda, Yulia Y. Djabir, Geoffrey P. Dobson

**Affiliations:** 1 Department of Physiology, Faculty of Medicine, Hasanuddin University, Makassar 90245, Indonesia; 2 Clinical Pharmacy Laboratory, Faculty of Pharmacy, Hasanuddin University, Makassar 90245, Indonesia; genikurnia07@gmail.com (G.K.R.L.); sesilialusiana8@gmail.com (S.L.L.); yulia.yusrini@unhas.ac.id (Y.Y.D.); 3 Heart, Trauma and Sepsis Research Laboratory, College of Medicine and Dentistry, James Cook University, Townsville, QLD 4811, Australia; geoffrey.dobson@jcu.edu.au

**Keywords:** adenosine, lidocaine, aorta, preservation solution

## Abstract

*Background and objectives:* The main problem of vascular preservation is the maintenance of vessel graft quality and function following extended storage. Conventional preservation solutions such as histidine–tryptophan–ketoglutarate (HTK) solution, Phosphate-Buffer Solution (PBS), or sodium chloride 0.9% has been shown to be inadequate in preserving vascular physiological function after 3 days of cold storage. This study aimed to evaluate whether adenosine and lidocaine (AL) in a modified Krebs–Henseleit (KH) solution can preserve the function and histological structure of rat aortic rings after 6 days. *Materials and Methods:* Thirty-five aortic rings from male Wistar rats (200–300 g) were harvested and immediately immersed in one of the assigned cold preservation solutions: standard KH, modified KH (mod KH) with lower calcium (Ca^2+^) and higher magnesium content (Mg^2+^) with or without adenosine and lidocaine (mod KH-AL), and modified KH with AL, insulin, and melatonin (Mod KH-ALMI). The contraction and relaxation function of the aortic rings were examined using an isometric force transducer after 6 days of cold preservation. Hematoxylin and eosin staining were used to analyze the rings’ histological structure. *Results:* Vascular contraction and relaxation functions were severely affected after a 6-day cold storage period in standard KH. Modifying the KH solution by reducing the Ca^2+^ and increasing the Mg^2+^ levels greatly recovered the vessel functions. The addition of AL or ALMI to the modified KH did not further recover vascular contractility. However, only the addition of AL to the modified KH increased the ACh-induced relaxation at 6 days when compared to the conventional KH, suggesting that endothelium preservation is improved. From histological analysis, it was found that the addition of AL but not ALMI further improved the endothelial lining and the structure of the elastic membrane layers of the preserved vessels after 6 days of cold preservation. *Conclusions:* The addition of AL to low calcium-high magnesium KH solution significantly enhanced endothelial preservation and improved endothelial-induced relaxation of preserved vessels after 6 days of cold storage.

## 1. Introduction

Small-diameter vessel grafts are regularly used in cardiovascular surgery to reconstruct dysfunctional or damaged blood vessels [1]. After being harvested, the vascular rings are often stored in an anti-spasm solution for several hours during the cardiovascular surgery [2], but prolonged storage for up to several days may require cold storage in a preservation solution [3]. The main problem of vascular preservation is the maintenance of vessel graft quality and function following prolonged storage [4]. Cold storage in conventional preservation solution, such as histidine–tryptophan–ketoglutarate (HTK) solution, Phosphate-Buffer Solution (PBS), or sodium chloride 0.9%, has been shown to be inadequate in preserving vascular contractility and relaxation after 3 days of storage [4]. Meanwhile, the standard cold preservation solution, Krebs–Henseleit, has been shown to preserve the contractility function of aortic grafts after 48 h of storage, but does not sufficiently preserve aortic relaxation function (30% loss of endothelial-dependent relaxation) [5]. 

The composition of the preservation solution plays pivotal roles in decelerating cell death mechanisms during cold storage. Preservation efficacy is limited by how each element contributes to halting molecular pathways that may progress to ischemic-/hypoxic-induced cellular injury. The constituents of the preservation solution are mostly adjusted to target specific pH, osmolality, osmolarity, nutrition, and often, antioxidative agents [6] to mimic the most ideal environment for the preserved tissues [7]. This could be varied due to cellular heterogeneity and organ complexity [8].

Endothelial injury is believed to be the key factor in vascular dysfunction following ischemic preservation, as endothelial cells are vulnerable to oxidative stress [9]. The deterioration of endothelial function can manifest as vascular occlusion, the increased risk of atherosclerosis and intimal hyperplasia, and reduced graft patency [10]. Therefore, maintaining endothelial viability during the cold preservation period is essential for improving the physiological function of vessel grafts and reducing morbidity following revascularization.

Adenosine is a well-recognized vasodilator with anti-inflammatory, antithrombotic, and cardioprotective properties [11,12]. Lidocaine is another vasoactive agent that offers anti-inflammatory and oxidative stress-prevention properties [13]. Lidocaine has also been shown to protect aortic endothelial and vascular smooth muscle (VSM) function [14,15]. As a local anesthetic agent, lidocaine also has a membrane-stabilizing effect that may preserve vascular reactivity [16]. Adenosine and lidocaine (AL) addition has been demonstrated to elicit superior anti-inflammatory, vasodilatory, and antiarrhythmic effects when compared with either drug alone [17,18,19]. AL combination with or without melatonin and insulin in Krebs–Henseleit solution with low Ca^2+^ and high Mg^2+^ (modified KH) has been shown to improve the function of isolated rat hearts as compared with the traditional preservation solutions Celsior or HTK following 8 h static cold storage [20]. Therefore, we hypothesized that this AL addition to a modified KH solution would improve the preservation ability of the solution on vascular function, and with melatonin and insulin addition would even further enhance the preservation. In this study, we aimed to investigate the effects of AL in a modified KH preservation solution, with and without melatonin and insulin, on rat aortic ring function and histology after 6 days of static cold preservation.

## 2. Materials and Methods

### 2.1. Chemicals

Adenosine, melatonin, and insulin were purchased from Sigma Aldrich (Singapore). Lidocaine HCl was obtained from Phapros TBK (Indonesia). All the salts used to prepare the Krebs–Henseleit solution were purchased from Merck (Singapore). All the solutions were prepared fresh daily before harvesting the aortic rings and stored in a refrigerator at 4 °C before starting the experiment.

### 2.2. Preservation Solution Compositions

- Krebs–Henseleit (KH): 118 mM NaCl, 4.7 mM KCl, 1.2 mM NaH_2_PO_4_, 2.5 mM CaCl_2_, 1.2 mM MgCl_2_, 25 mM NaHCO_3_, 0.03 mM Na-EDTA, and 11 mM glucose.
- Modified KH: 118 mM NaCl, 4.7 mM KCl, 1.2 mM NaH_2_PO_4_, 0.22 mM CaCl_2_, 2.6 mM MgCl_2_, 25 mM NaHCO_3_, 0.03 mM Na-EDTA, and 11 mM glucose.
- Modified KH + AL: modified KH, 0.4 mM adenosine, and 1 mM lidocaine.
- Modified KH +ALMI: modified KH, 0.4 mM adenosine, 1 mM lidocaine, 0.1 mM melatonin, and 0.01 IU/mL insulin.

Melatonin and insulin were incorporated into the AL solution for the following reasons: (1) Melatonin has free radical scavenging and antioxidative actions. Due to its lipophilic nature, melatonin can easily enter the cell, reach organelles and the nucleus, and protect them against oxidative damage [21,22]. (2) Insulin is a potent K^+^ influx enhancer, which may have the ability to restore intracellular K^+^ levels and initiate membrane hyperpolarization, as observed in skeletal muscle [23]. Additionally, insulin can improve glucose uptake and utilization, hence, improving ATP production.

### 2.3. Animal Preparation

Thirty-five male Wistar rats (200–300 g) were cared for in a laboratory with a 12 h light–dark cycle. The rats were provided with food and water ad libitum. All the animal protocols were performed based on the Guide for the Care and Use of Laboratory Animals and were granted ethical clearance by Hasanuddin University (224/UN4.6.4.5.31/PP36/2022). The rats were anesthetized with ether inhalation before performing the surgical protocols.

### 2.4. Aortic Ring Preparation

The rat thoracic aorta vessels were harvested and immediately immersed in cold KH solution. The vessel segments were carefully cleaned from the surrounding adipose and connective tissue and were cut in 3 mm length rings as previously described in a study by Arsyad [18]. The fresh aortic rings, serving as the control group, were directly mounted on small stainless-steel triangular stirrups in an organ bath filled with KH solution and rewarmed to 37 °C for vessel function analysis. Meanwhile, the aortic segments assigned to the other groups were stored for 6 days in one of the following cold solutions: Krebs–Henseleit (KH), modified KH (low Ca^2+^/high Mg^2+^), modified KH with adenosine and lidocaine (KH + AL), and modified KH with adenosine, lidocaine, melatonin, and insulin (KH + ALMI). Based on a previous study on porcine, arterial wall structure may deteriorate after six days of preservation in cold storage using a standard solution [24]. The solution containing melatonin was covered with aluminum foil during storage to prevent oxidation. 

### 2.5. Vessel Function Analysis

The physiological function of the aortic rings was measured using an isometric force transducer (ADInstruments) coupled with a data acquisition system and software (ADInstruments Pty Ltd., Sydney, Australia) as described in a previous study by Arsyad [18]. Apart from the control aortic rings, all the other rings were stored in the assigned cold solution for 6 days. Following a 6-day static cold period, the aortic rings were gently rewarmed to 37 °C in an organ bath containing the modified KH solution (pH 7.4) and constantly aerated with 95% O_2_ and 5% CO_2_. After 15 min of equilibration period (zero tension), the rings were vertically mounted on a small stainless stirrup that was connected to an isometric force transducer. The ring tension was manually adjusted to 1.5 g for a duration of 60 min [25]. The contraction was induced by norepinephrine (0.3 μM) and a high-potassium saline solution (60 mM KCl) to observe the receptor- and non-receptor-induced contractile responses. 

Acetylcholine (ACh) and sodium-nitroprusside (NO donor) relaxation effects were tested to assess the endothelium-dependent and -independent relaxation, respectively. In the presence of endothelium, ACh induces an increase in intracellular calcium concentration and leads to the production and release of NO and prostacyclin (PGI_2_) in endothelial cells in addition to the opening of K^+^ channels, which in turn induces vascular smooth muscle cell relaxation [26]. SNP was utilized as an endothelium-independent vasodilator due to its primarily direct effect on smooth muscle cells by releasing NO into the vascular smooth muscle cell without requiring enzymatic conversion [27]. At the end of each experiment, the rings were maximally dilated with 100 μM papaverine to confirm the viability of the vessels and to obtain the maximum relaxation value. The vessel function measurements were described in detail in our previous studies [13,25].

### 2.6. Vessel Histopathological Analysis

**Figure 1:**
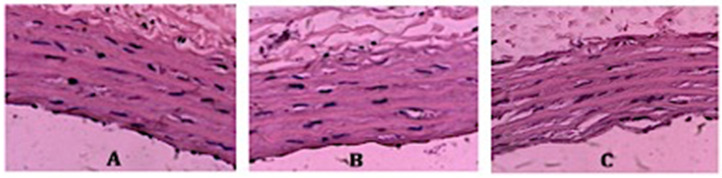
Representative vascular sections with degeneration scores of 0 (**A**), 1 (**B**), and 2 (**C**).

At the end of each preservation period, the aortic ring was fixed with 10% (*v*/*v*) formalin and processed to obtain vascular histological slides. The slides were then stained with hematoxylin and eosin (H&E) for histopathological analysis and assessed under a light microscope (Olympus) by experienced pathologists. The endothelial cells were identified as a single layer showing a single layer of squamous-fusiform cells lining the lumen of the vessels [28]. The degree of damage was also considered. The criteria of histopathological vessel damage included elastic membrane ablation, endothelial denudation, and tunica intima layer degradation [29]. The severity of vascular degeneration was scored according to a study by Kongpol, et al. (2020): score 0, no change or minimal vascular degeneration; score 1, vascular degeneration observed in less than 25% of the observed area; and score 2, vascular degeneration observed in more than 25% of the observed area [28]. Representative vascular sections with degeneration scores of 0 to 2 are provided in Figure 1.

### 2.7. Statistical Analysis

All the values were expressed as mean ± SEM. The power of statistical analysis was determined by a G*Power program (version 3.1.9.6). A priori power for repeated measures between the factors for ANOVA analysis was calculated based on 7 measurements and 5 treatment groups with a 95% confidence interval. The calculation showed a sample size of 7 per group obtained an actual power of 1.00. The values of contraction were reported in gram tension while the relaxation responses were reported as a percentage of the maximal relaxation to 100 µM papaverine. All the data were analyzed with Kolmogorov–Smirnov test to determine the normal distribution. Significant differences among the treatment groups were analyzed using one-way ANOVA followed by a Bonferroni post hoc test to compare the individual data points.

## 3. Results

### 3.1. NE- and KCl-Induced Aortic Vasoconstrictive Responses with Different Preservation Solutions

**Figure 2:**
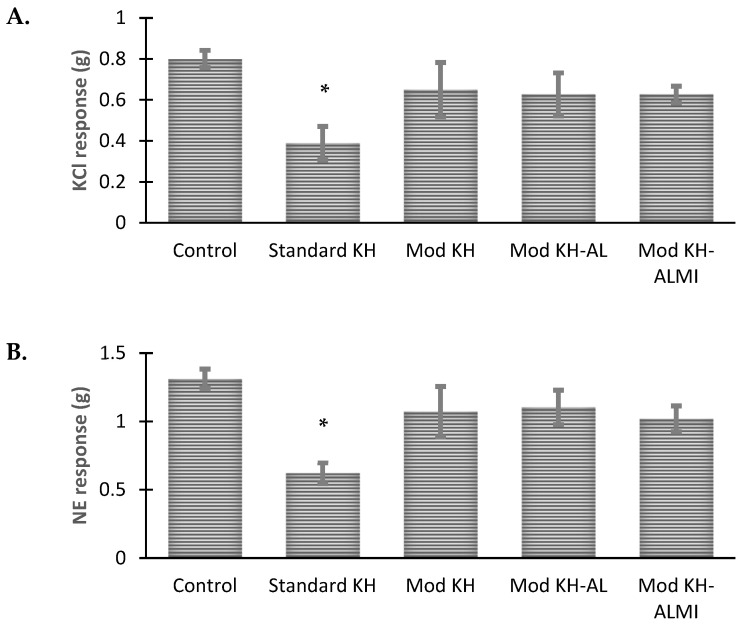
Isolated rat aortic ring contraction after 6 days of cold preservation compared to the fresh preparation (control). Response to potassium chloride (KCl) (**A**) and norepinephrine (NE) (**B**). The values represent the mean ± S.E.M of the aortic ring contraction. * *p* < 0.05 between the standard KH and control groups.

The freshly harvested aortic ring (control) produced vasoconstriction after the administration of KCl and NE, with contractility responses of 0.80 ± 0.42 and 1.31 ± 0.07 g tension, respectively (Figure 2). After 6 days of storage in the KH solution at 4 °C, the aortic rings produced only 0.39 ± 0.08 and 0.62 ± 0.08 g tension in response to KCl and NE, respectively; less than 50% of the contraction produced by the freshly harvested aortic rings (controls). Differing from the rings preserved in the standard KH solution, when the solution was modified by decreasing the concentration of Ca^2+^ and increasing Mg^2+^ (modified KH solution), the contraction of the aortic rings was maintained and was equivalent to ~80% of the control aortic rings. In this group, the aortic contractions with KCL and NE were 0.865 ± 0.13 and 1.07 ± 0.19 g tension, respectively. 

Meanwhile, preservation in the modified KH solution with AL and ALMI sustained aortic contraction following the administration of KCl and NE at 84% and 78%, respectively. These results were not significantly different from the aortic contraction response of the rings stored in the modified KH solution only (78%). This indicated that the modification of KH by lowering the concentration of Ca^2+^ and increasing Mg^2+^ was effective in improving the contractile function of the aortic smooth muscle with or without the addition of AL or ALMI (Figure 2).

### 3.2. Ach- and NO-Induced Aortic Vasorelaxation Responses in Different Preservation Solutions

To examine the endothelium-dependent and -independent vasodilative function, the relaxation response to acetylcholine (ACh) and nitric oxide (NO) donor (sodium nitroprusside) of the aortic rings stored in the preservation solutions was measured and compared with papaverine. The relaxation response is formulated as a percent of the relaxation to 100 μM papaverine.

**Figure 3:**
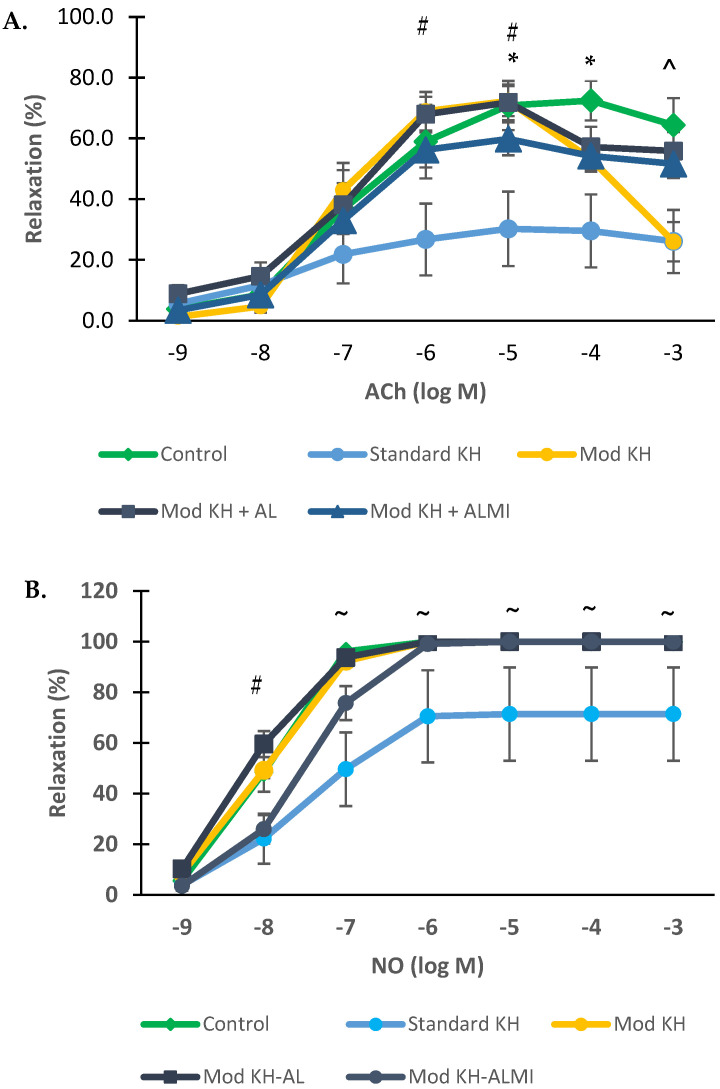
Isolated rat aortic ring percent relaxation after 6 days of cold preservation. Response to acetylcholine (**A**) and nitric oxide donor (**B**) compared to the control. Relaxation is expressed as the percent of relaxation to 100 μM papaverine. The points represent the mean ± S.E.M of the aortic ring relaxation. * *p* < 0.05 between the control and standard KH groups. # *p* < 0.05 between the standard KH and modified KH−AL groups. ^ *p* < 0.05 between the modified KH and modified KH−AL and KH−ALMI groups. ~ *p* < 0.05 between the KH and the other groups.

The freshly harvested aortic ring (control) produced a relaxation response to Ach with the percentages of 3.8 ± 1.5%, 8.6 ± 2.9%, 36.7 ± 15.27%, 58.9 ± 12.09%, 70.8 ± 8.08%, 72.4 ± 6.51%, and 64.4 ± 8.87% for the ACh concentrations of 10^−9^, 10^−8^, 10^−7^, 10^−6^, 10^−5^, 10^−4^, and 10^−3^ M, respectively (Figure 3). After preserving in the standard KH solution for 6 days, the aortic relaxation response was significantly reduced by 21.8 ± 9.80% at 10^−5^ M ACh and 30.2 ± 12.28% at 10^−4^ M ACh, and was significantly different when compared with the controls. In fact, three out of eight aortic rings stored for 6 days in the standard KH solution produced no response to the ACh stimulation.

When stored in the modified KH solution with low Ca^2+^ and high Mg^2+^, the aortic relaxation significantly increased to 68.9 ± 6.36% and 72.3 ± 4.97% at the ACh concentrations of 10^−6^ and 10^−5^ M, respectively. These values were significantly higher than those with the standard KH at the ACh concentrations of 10^−6^ and 10^−5^ M (*p* < 0.05). However, the relaxation response of the aortic rings preserved in the modified KH solution noticeably decreased to 26.0% after the ACh concentration was increased to 10^−3^ M. The addition of AL to the modified KH solution allowed the maintenance of the relaxation response at the ACh concentrations of 10^−6^ to 10^−5^ M and persisted above 50% relaxation at an ACh concentration of 10^−3^ M. When compared with the modified KH, the aortic rings stored in the KH with AL or ALMI had significantly improved relaxation responses to an ACh concentration of 10^−3^ M (*p* < 0.05) (Figure 3A).

The relaxation percentage of the control aortic ring in response to NO is shown in Figure 3B. At the lowest concentration of NO (10^−9^ M), the relaxation started to occur at 5.6 ± 1.40% and continued to increase until it reached 100% at the concentrations of 10^6^ to 10^−3^ M. After 6 days of storage in the KH solution, the aortic relaxation response to NO was significantly decreased as compared with the controls, with a maximum relaxation of 71 ± 18.44% at the concentrations of 10^−5^ to 10^−3^ M. In contrast, the use of the modified KH with or without AL resulted in relaxation responses to NO of 100%, which were not significantly different from the controls regardless of prolonged storage (6 days) in the preservation solution. However, the addition of ALMI slightly reduced the relaxation response to NO compared with the modified KH alone at the NO concentrations of 10^−8^ to 10^−7^ M. All three modified KH solutions (with or without AL or ALMI) caused 100% relaxation starting from the NO concentration of 10^−5^ M (Figure 3B).

### 3.3. Maximum Aortic Relaxation Function after 6 Days of Storage in Preservation Solution Compared with Freshly Harvested Aortic Rings

**Figure 4:**
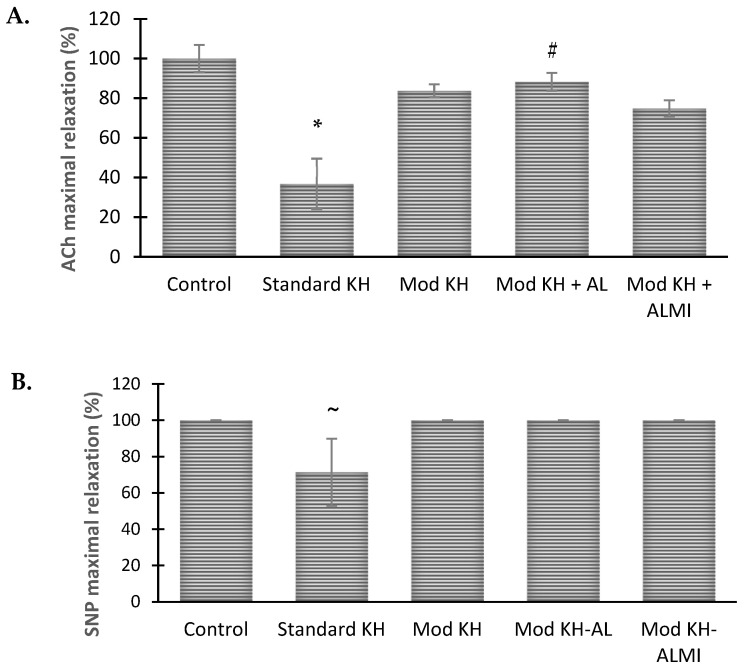
Concentration–response curve for SNP (NO-induced relaxation) after 6-day storage compared to control. (**A**) Acetylcholine-induced maximal relaxation, and (**B**) SNP-induced maximal relaxation. Relaxation is expressed as the percent of the control group maximal relaxation. The values are expressed as the mean ± S.E.M of the aortic ring relaxation. * *p* < 0.05, statistical difference between the KH and the control group. # *p* < 0.05, the KH group statistically differs from the modified KH + AL groups. ~ *p* < 0.05 between the KH and the other groups.

The maximum relaxation achieved by the control aortic rings was 85.4% in response to ACh, and thus, this value was used as the standard of maximum relaxation (100%). After 6 days of standard KH storage, the ACh-dependent relaxation of the aortic ring was significantly altered, where the rings obtained a maximum relaxation of only 36.8 ± 12.7% (Figure 4A). The modified KH preservation solution improved relaxation recovery after 6 days of storage and attained a maximum response of 83.8 ± 3.3%. The maximum relaxation of the rings somewhat increased with the addition of AL as compared with the modified KH alone, which achieved a maximum relaxation of 88.3 ± 4.5%. In contrast, the addition of ALMI to the modified KH did not increase and even reduced the maximum relaxation to ACh, and provided only 74.8 ± 4.1% aortic recovery after 6 days in cold storage (Figure 4A). Meanwhile, as with the controls, the aortic rings preserved in any of the three modified KH solutions (without or with AL or ALMI) could reach a maximum relaxation of 100% in response to NO (Figure 4B).

### 3.4. Vessel Histopathological Analysis after 6 Days of Storage in Preservation Solution

**Figure 5:**
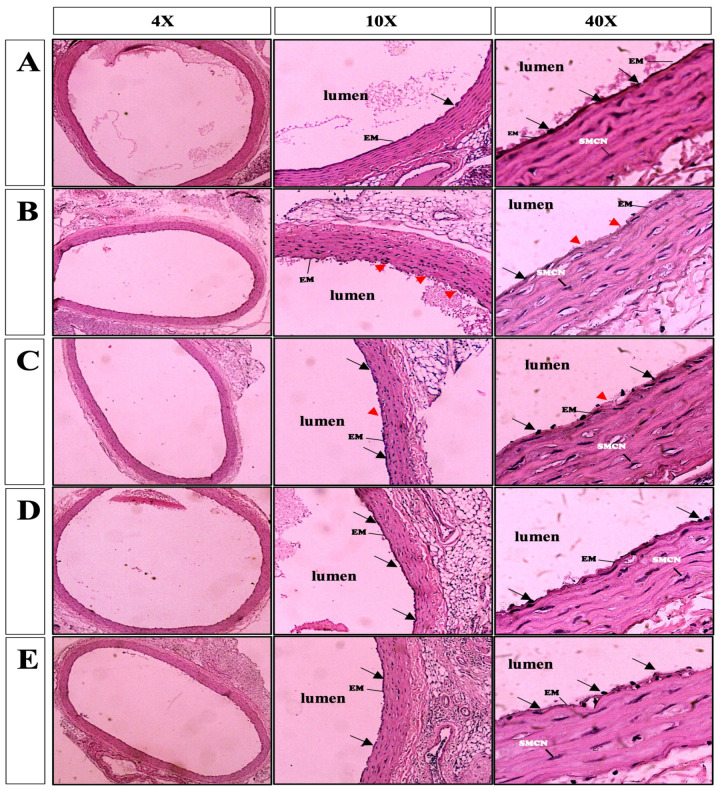
The representative of the hematoxylin and eosin section of freshly harvested aorta (**A**) and after 6-day preservation in the KH (**B**), modified KH (**C**), modified KH-AL (**D**), and modified KH-ALMI solution (**E**) in different microscopic magnifications. The black arrows show the endothelial cells; the red arrowheads show the denudation of the endothelium; EM: elastic membrane; SMCN: smooth muscle cell nucleus.

In freshly harvested aortic rings, the endothelium, tunica intima layers, and adventitia showed normal architecture (Figure 5A, 4×, 10×, 40×). The aortic vessels stored in the standard KH for 6 days showed almost-complete denudation of the endothelial cells and elastic membrane deterioration (Figure 5B, 10×, 40×), while the aortic rings stored in the modified KH solution exhibited a significantly lower grade of endothelial detachment and elastic membrane disruption when compared with the KH group (Figure 5C, 10×, 40×). Furthermore, the vessels maintained in the modified KH with AL showed more improved vessel structure, especially at the endothelial lining of the vascular lumen, as compared with the vessels maintained in the modified KH only (Figure 5D, 10×, 40×). The addition of ALMI produced no significant difference in elastic membrane deterioration grade when compared with those preserved in the modified KH solution (Figure 5E, 10×, 40×).

The severity of vascular degeneration was scored based on the presence of elastic membrane deterioration and the denudation of the endothelial cells observed (Table 1). It was found that freshly harvested aorta had no substantial vascular degeneration. In contrast, the damage found in the vessels preserved in the KH solution for 6 days affected more than 50% of the observed area (score 2). The presence of histopathological changes was reduced in the vessels stored in the modified KH solution (score 1) and was even lesser when stored in the modified KH-AL and KH-ALMI solutions (score 0). 

## 4. Discussion

Maintaining the physiological and biochemical properties of the preserved vessel grafts is of importance during vessel banking to prevent dysfunctional vessels following revascularization [30]. Tissue or organ preservation solutions mostly contain some basic components such as impermeants (glucose, raffinose, mannitol, lactobionate, etc.) and electrolytes (sodium, chloride, potassium, calcium, magnesium, phosphate, bicarbonates, sulfates, etc.) to provide proper osmolality and pH in the solution. Currently, commercial preservation solutions are often enriched with antioxidants, nutrients, and specific additives (insulin, antibiotics) that may protect against pathological molecular responses induced by ischemia and hypoxia [7,8]. 

In this study, adenosine and lidocaine, with or without insulin and melatonin, were incorporated in a KH solution to improve its preservation efficacy during prolonged storage. It is shown that after 6 days of cold preservation, the contractility function of the aortic rings stored in the standard KH was significantly reduced in response to NE and KCl, respectively. This finding confirmed that the standard KH solution failed to prevent impairment in the adrenergic receptor-stimulated and ion channel-dependent contraction of the aortic rings [31]. However, this impairment can be improved by reducing the Ca^2+^/Mg^2+^ ratio of the solution with or without the addition of AL and ALMI. 

Ingemansson et al. (1995) reported that standard KH may restore around ~90% of vascular contractility within 36 h of preservation. The maintenance of aortic contractile function was thought to be derived from the calcium content [32]. Similarly, the addition of Ca^2+^ (1.5 mM) to calcium-free solutions such as UW, Perfadex, and Euro-Collins solution also contributed to the recovery of aortic contractile function after storage for 24 to 36 h [33,34]. However, following prolonged storage (144 h), a normocalcemic solution (1.5–2.5 mM Ca^2+^), as in the standard KH, was found unfavorable for maintaining vascular contractile function.

It is widely recognized that Ca^2+^ ions play a crucial role in the regulation of cell death. An extracellular fluid with high levels of Ca^2+^ ions can trigger the influx of Ca^2+^ ions into cells, leading to intracellular Ca^2+^ overload and ultimately inducing cell apoptosis [35]. Therefore, reducing the concentration of Ca^2+^ in a solution may mitigate the negative effects of Ca^2+^ ions. Additionally, high levels of Mg^2+^ ions have been found to counteract the harmful effects of elevated Ca^2+^ ion levels in cells. A study demonstrated that Mg^2+^ acts as a specific type of Ca^2+^ channel antagonist in VSM [36]. Furthermore, another study revealed that the reperfusion of the heart with high Mg^2+^ levels reduces mitochondrial Ca^2+^ overload and helps maintain mitochondrial ATP production capacity. This study also indicated that lowering calcium content in the presence of high magnesium leads to better protection [37]. These findings suggest that decreasing Ca^2+^ concentration and increasing Mg^2+^ concentration in a preservation solution may yield promising results in prolonging cell survival. 

Lowering the Ca^2+^ concentration to 0.2 mM (in addition to increasing the Mg^2+^ content) in the KH solution not only increased contractility response, but also was superior in enhancing the vasodilation response to ACh and NO. The vasodilation response to ACh and NO represents endothelium-dependent and -independent relaxation function [38]. In this study, aortic preservation in the standard KH caused 63% and 29% loss of endothelium-dependent and -independent responses, respectively. These results support those of the previous studies by Ingemansson and colleagues, who showed that although standard KH could recover rat infrarenal aortic contractile function, the maintenance of the relaxation function was unsuccessful [32,33,34]. This finding implies that a high concentration of Ca^2+^ is harmful to endothelial function, and that decreasing the Ca^2+^ and increasing the Mg^2+^ concentration is valuable not only in preventing the loss of endothelial-dependent relaxation, but also in maintaining endothelium-independent vasodilation.

After 6 days of cold storage, endothelial detachment was observed in the rings preserved in the standard KH solution (Figure 5B), in which the endothelial detachment scores predominated. Usually, endothelial cell shedding occurs within 48 h of preservation, and mainly necrotic cells disappear from the vessels; in this case, fewer necrotic cells were observed after 6 days [29]. Since an intact luminal endothelial barrier is imperative to graft patency [39], the addition of AL, with or without melatonin and insulin, in a modified KH solution (lower Ca^2+^/Mg^2+^ ratio) may not only improve the preservation of the vessel architecture but also potentially improve vascular graft patency and function. 

It has been shown that the AL solution significantly increased endothelial nitric oxide synthase (eNOS) dimerization, maintained the endothelial glycocalyx, and promoted pro-survival signaling as seen in coronary cardiac grafts [40]. During cold storage, the graft shall experience the depletion of adenosine triphosphate (ATP) cellular stores and the inhibition of Na^+^/K^+^ATPase, leading to a drop in the intracellular K^+^ levels and membrane depolarization, increasing the production of reactive oxygen species (ROS) [41]. Lidocaine, through its activation of mitochondrial K_ATP_ channels, was able to diminish cytokine-induced injury in endothelial and VSM cells [14]. In combination, AL has been shown to decrease oxidative injury and intracellular calcium overload in preserved cardiac grafts [40]. The protective effects of AL on endothelial cells may be linked to its capacity to restore and protect the endothelial glycocalyx [40,42]. Adenosine in preservation solution may allow the rapid regeneration of energy (ATP) [7] while providing other benefits such as the inhibition of endothelial inflammation [43]. The membrane-stabilizing effect of lidocaine may protect against cellular reactivity. Moreover, pretreatment with lidocaine has been shown to provide protection against ROS-induced endothelial dysfunction in rabbit aortic rings [44]. 

Additionally, adenosine and lidocaine may independently influence the cellular pathways responsible for vascular contractility and relaxation. It was found that through the A2a receptor, adenosine elicits vasodilation by releasing endothelial NO and stimulating the opening of VSM K_ATP_ channels [45] whilst lidocaine inhibits Ca^2+^ entry through voltage- and receptor-gated Ca^2+^ channels, and reduces Ca^2+^ release from intracellular stores, thereby reducing the intracellular free Ca^2+^ that is necessary for the contraction of VSM [46].

Meanwhile, the addition of melatonin and insulin as hormone and trophic factors may act as anti-inflammatory, antioxidant, and anti-apoptotic agents [47]. Melatonin has been found to improve organ transplantation and reduce graft failure when added to a range of preservation solutions, including University of Wisconsin (UW) and histidine–tryptophan–ketoglutarate (HTK) [48,49]. These effects may add extra protection against ischemic reperfusion injury.

At the moment, DuraGraft^®^ is the only preservation solution that has been clinically approved as a vascular preservation solution [50]. However, the efficacy of DuraGraft^®^ as a preservation solution is limited to protect saphenous vein grafts during intra-operative intervals up to 24 h [51,52,53]. There is still an unmatched demand for preservation solutions for prolonged ischemic storage. The results of this study may provide new insights into adding AL and lowering the Ca^2+^/Mg^2+^ ratio of KH solution to potentially improve vascular graft patency and function following extended cold storage.

### Limitation of the Study

This study has several limitations. Firstly, we used aortic rings as the model for vascular graft preservation, whereas clinical settings typically utilize small arterial or venous segments for bypass surgery. Therefore, the results of our study may have discrepancies if different conduits are used. Secondly, the KH preservation solutions were freshly prepared daily, which may not be feasible in clinical settings. Thirdly, for histological evaluation, we only employed H&E staining to visualize the structural integrity of the endothelium and VSMCs. Incorporating immunohistochemistry staining could be beneficial for detecting endothelial nitric oxide synthase (eNOS) protein. 

## 5. Conclusions

Vascular contraction and relaxation functions were severely affected after a 6-day cold storage period in normal KH. Modifying the KH solution by reducing the Ca^2+^ and increasing Mg^2+^ levels greatly recovered the vessel functions. The addition of AL or ALMI to the modified KH did not further recover vascular contractility. However, only the addition of AL to the modified KH increased ACh-induced relaxation at 6 days when compared to the conventional KH, suggesting that endothelium preservation is improved. From the histological analysis, it was found that the addition of AL but not ALMI further improved the endothelial lining and the structure of the elastic membrane layers of the preserved vessels after 6 days of cold preservation.

## Figures and Tables

**Table 1:** The score of vascular degeneration after 6 days of cold storage in KH, modified KH, modified KH-AL, and modified KH-ALMI.

Groups	Elastic Membrane Deterioration	Denudation of Endothelial Cells
Control	0	0
Standard KH	2	2
Mod KH	1	1
Mod KH-AL	0	0
Mod KH-ALMI	0	0

0 = no change or minimal degeneration; 1 = <25% degeneration; 2 = >25% degeneration.

## Data Availability

The data that support the findings of this study are not publicly available due to confidentiality agreements.
